# Cardiovascular Outcomes with Colchicine in Coronary Artery Disease and HFpEF: A Propensity-Matched TriNetX Analysis

**DOI:** 10.3390/jcdd13060222

**Published:** 2026-05-23

**Authors:** Faizan Ahmed, Saifullah Khan, Madeeha Shafqat, Najam Gohar, Muhammad Hassan, Muhammad Hussain, Haris Bin Tahir, Tehmasp Rehman Mirza, Muhammad Abdullah, Syed Muhammad Murtaza Ishaq, Haider Hussain Shah, Mohammad Hamza, Mohammad Omar Butt, Fenilkumar Kotadiya, Amro Taha, Swapnil Patel, Fawaz Alenezi

**Affiliations:** 1 Department of Internal Medicine, Jersey Shore University Medical Center, Neptune, NJ 07753, USA; swapnil.patel@hmhn.org; 2 Deparment of Internal Medicine, Dow Medical College, Dow University of Health Sciences, Karachi 74200, Pakistan; saifullah.khan23@dmc.duhs.edu.pk (S.K.); muhammad.hassan23@dmc.duhs.edu.pk (M.H.); muhammad.hussain23@dmc.duhs.edu.pk (M.H.); 3 Department of Internal Medicine, Geisinger Medical Center, Danville, PA 17822, USA; mshafqat@geisinger.edu; 4 Department of Internal Medicine, Ameeruddin Medical College, Post Graduate Medical Institute (PGMI), Lahore 54000, Pakistan; najamgoharr@gmail.com; 5 Department of Internal Medicine, Lahore General Hospital, Lahore 54000, Pakistan; harristahirchh@gmail.com; 6 Department of Internal Medicine, Shalamar Medical and Dental College, Lahore 54000, Pakistan; mbbs2023137@student.smdc.edu.pk (T.R.M.); mohd_abdullah2003@outlook.com (M.A.); 7 Department of Internal Medicine, SUNY Upstate Medical University, Syracuse, NY 13210, USA; ishaqs@upstate.edu; 8 Department of Internal Medicine, Bayhealth Hospital, Kent Campus, Dover, DE 19901, USA; haider.shah@apogeephysicians.com; 9 Department of Internal Medicine, Guthrie Medical Group, Cortland, NY 13045, USA; mohammad.hamza@guthrie.org; 10 Department of Internal Medicine, Memorial Satilla Health, Waycross, GA 31501, USA; mohammadomar_butt@teamhealth.com; 11 Department of Internal Medicine, Charleston Area Medical Center, Charleston, WV 25304, USA; fenilkumar.kotadiya@camc.org; 12 Division of Cardiology, West Virginia University, Morgantown, WV 26506, USA; taha61591@gmail.com; 13 Division of Cardiology, Duke University School of Medicine, Durham, NC 27710, USA; fawaz.enezi@duke.edu

**Keywords:** colchicine, coronary artery disease, HFpEF, heart failure with preserved ejection fraction, inflammation, propensity score matching, cardiovascular outcomes, TriNetX

## Abstract

Background: Coronary artery disease (CAD) and heart failure with preserved ejection fraction (HFpEF) are major causes of morbidity and mortality. Systemic inflammation contributes to both, suggesting potential benefit from colchicine, though data in CAD-HFpEF are limited. Methods: We conducted a real-world study using the TriNetX Research Network, identifying 480,434 adults with CAD and HFpEF. Patients were categorized as colchicine users (*n* = 30,254) or non-users (*n* = 450,180). One-to-one propensity score matching yielded 28,941 patients per group. The primary outcome was a composite of acute myocardial infarction, stroke, all-cause mortality, and acute heart failure. Secondary outcomes included individual components, hospitalizations, atrial fibrillation, and gastrointestinal events, assessed at 1- and 3-year follow-up. Results: At 1 year, the primary outcome occurred in 16.9% of the colchicine group versus 19.8% of non-users (HR: 0.86, 95% CI: 0.81–0.91; *p* < 0.001). All-cause mortality was 11.4% versus 14.5% (HR: 0.77, 95% CI: 0.73–0.80; *p* < 0.001). At 3 years, the primary outcome occurred in 34.2% versus 39.7% (HR: 0.88, 95% CI: 0.85–0.92; *p* < 0.001), with acute heart failure slightly higher in the colchicine group (27.5% vs. 26.5%; HR: 1.04, 95% CI: 1.01–1.08; *p* = 0.009). Stroke was modestly reduced (HR: 0.93, 95% CI: 0.88–0.99; *p* = 0.014). No significant differences were seen in all-cause hospitalization, atrial fibrillation, or gastrointestinal events. Conclusions: In patients with CAD and HFpEF, colchicine was associated with lower short- and medium-term risk of composite cardiovascular events and mortality, with modest attenuation over time, suggesting early time-dependent association rather than sustained structural effects. Prospective randomized trials are warranted.

## 1. Introduction

Cardiovascular diseases (CVDs), including CAD and HfpEF, represent the leading causes of morbidity and mortality worldwide, placing significant strain on healthcare systems. HFpEF is a growing and complex condition, particularly among individuals with pre-existing CAD. Recent studies suggest that systemic inflammation is a key factor in the development of both CAD and HFpEF, highlighting its potential as a therapeutic target [1,2]. A pooled analysis of prospective studies on HFpEF indicates that around 50% of patients with this condition have coexisting CAD [3]. Colchicine, a well-known anti-inflammatory drug traditionally used in the treatment of gout, has recently gained interest for its ability to reduce inflammation and potentially enhance cardiovascular outcomes. However, its impact on individuals with CAD and HFpEF remains insufficiently studied.

The current literature suggests that colchicine is associated with improved cardiovascular outcomes in prior studies as an anti-inflammatory agent for cardiovascular conditions such as CAD, with growing interest in its potential role in HFpEF due to shared inflammatory pathways; however, the evidence remains preliminary and indirect [4,5]. Most HFpEF trials exclude patients with significant CAD or recent ACS, leaving the interaction between CAD and HFpEF underexplored. Additionally, these studies rely on surrogate markers rather than hard endpoints, limiting their real-world applicability. The lack of large-scale randomized controlled trials (RCTs) and real-world data further restricts generalizability. Larger, more inclusive studies are needed to address these gaps and improve treatment strategies.

Our study uses a large real-world cohort from the TriNetX Research Network to evaluate associations between colchicine use and cardiovascular outcomes in patients with coexisting coronary artery disease (CAD) and heart failure with preserved ejection fraction (HFpEF). Unlike prior randomized trials in stable or post-myocardial infarction populations, data specifically addressing this combined phenotype remain limited. By leveraging propensity score matching across a broad multicenter dataset, this analysis aims to explore potential associations between colchicine exposure and clinical outcomes, including mortality and cardiovascular events, in a real-world setting. Given the observational nature of this study, the findings should be interpreted as hypothesis-generating and do not establish causality or inform guideline-directed therapy. Further prospective randomized studies are required to validate these associations in well-phenotyped HFpEF populations.

## 2. Methods

### 2.1. Data Source

**Figure 1:**
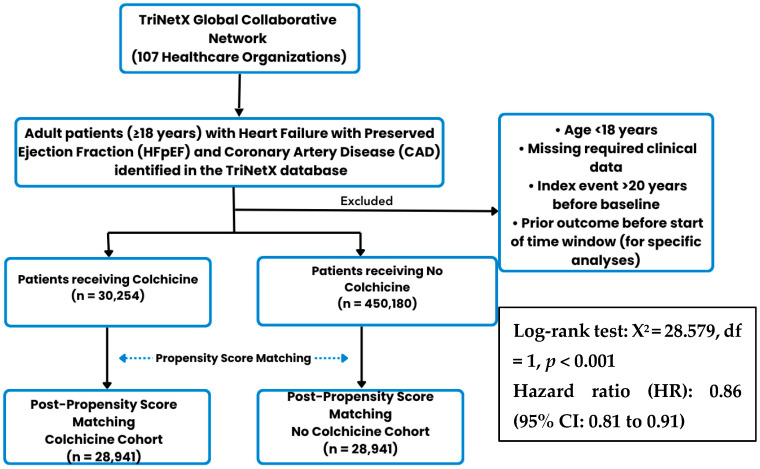
Study flow diagram.

This real-world study was conducted using the TriNetX Research Network, a global federated health research network which provides access to de-identified electronic medical records (EMRs). The query for this analysis was generated on 23 February 2026, which consists of clinical data from 107 diverse healthcare organizations (HCOs) (Figure 1). The TriNetX Network aggregates and de-identifies patient-level data which includes diagnoses, procedures, medications, and laboratory values in compliance with the Health Insurance Portability and Accountability Act (HIPAA). As this study used only de-identified, aggregated data, it was exempt from institutional review board approval per institutional policy.

### 2.2. Study Population and Design

Patients aged 18 years or older with both CAD and HFpEF were identified. HFpEF was identified using ICD-9 and ICD-10 codes for diastolic heart failure. CAD was identified using a comprehensive list of diagnostic codes for coronary atherosclerosis, myocardial infarction, and other forms of chronic or acute ischemic heart disease. The complete list of ICD-9 and ICD-10 codes used to define HFpEF and CAD is provided in the Supplementary Files.

Two cohorts were established based on colchicine use. Exposure was modeled as a time-fixed variable based on documented colchicine prescription records occurring at or after the combined diagnosis of CAD and HFpEF. Because TriNetX does not support time-varying exposure modeling, changes in colchicine use over time, including delayed initiation, discontinuation, variable adherence, intermittent use, or crossover exposure, could not be accounted for. The colchicine cohort (Cohort A, n = 30,254) included patients receiving colchicine (RxNorm: 2683) at the time of or after the diagnosis of CAD and HfpEF (Supplemental Table S1). The no-colchicine cohort (Cohort B, n = 450,180) included patients with the same diagnoses who never received colchicine (Supplemental Table S2). The index event for each patient was defined as the date they first met the combined diagnosis and medication criteria. Patients whose index event occurred more than 20 years ago were excluded. Outcomes were analyzed across two follow-up windows: a 1-year window (30 to 365 days post-index) and a 3-year window (30 to 1095 days post-index). To partially mitigate immortal time bias and ensure that exposure classification preceded outcome assessment, a 30-day landmark period was applied, with follow-up beginning 30 days after the index date.

### 2.3. Study Endpoints

The primary study endpoints included a primary composite outcome (comprising acute myocardial infarction [AMI], stroke [CVA], all-cause mortality, and acute heart failure [diastolic decompensation]); acute heart failure events were identified using ICD-10 codes I50.31 (acute diastolic congestive heart failure) and I50.33 (acute on chronic diastolic congestive heart failure). Secondary outcomes included all-cause mortality, all-cause hospitalization or emergency department (ED) visits, stroke (I60–I69), acute heart failure (I50.31, I50.33), atrial fibrillation, and gastrointestinal (GI) symptoms (R10–R19) (safety outcome) (Supplemental Tables S3 and S5). All-cause mortality was determined by the vital status code “deceased”. Hospitalizations and ED visits were identified using standardized visit-type codes including emergency (EMER), inpatient acute (ACUTE), and inpatient encounter (IMP). Patients with a given outcome prior to the start of the time window were excluded from that outcome analysis. Therefore, outcome-specific sample sizes vary depending on data availability and prior events, as reflected in the Results Section. For all-cause mortality and the primary composite outcome, patients with the outcome prior to the start of the time window were excluded. For each time-to-event analysis, TriNetX automatically excluded patients with a documented history of the corresponding outcome prior to the index event to assess incident events only. Accordingly, denominator populations differed across outcomes based on baseline disease prevalence. Additionally, eligible populations occasionally differed between 1-year and 3-year analyses because TriNetX independently generated survival cohorts for each follow-up interval according to platform-specific cohort construction and data availability criteria.

### 2.4. Statistical Analysis

All analyses were conducted on 1:1 propensity score-matching (PSM) cohorts, resulting in balanced groups of 28,941 patients each. To minimize baseline imbalances, 1:1 PSM was performed using greedy nearest-neighbor matching with a caliper of 0.1 times the pooled standard deviation (SD) of the linear propensity scores [6,7]. Matching was performed on 67 characteristics, including demographics (age at index, sex, and race), laboratory values (HbA1c, BNP, and GFR), and a wide array of comorbid diagnoses and medications (Supplementary Figure S1). The complete list of propensity score-matched variables is provided in Supplemental Tables S4 and S6.

Incidence rates and relative risks (RRs) with 95% confidence intervals (CIs) were estimated using the TriNetX Compare Outcomes Analysis model. Time-to-event outcomes were examined using Kaplan–Meier survival curves with daily time intervals, and differences between groups were evaluated using the log-rank test [8]. Cox proportional hazards regression models were used to measure hazard ratios (HRs) and 95% CIs [9]. Statistical significance for all analyses was defined as a two-sided *p*-value < 0.05. Outcome-specific analyses used varying denominators due to exclusion of patients with prior events before the start of each time window. Additionally, a sensitivity analysis was performed in a restricted cohort of patients with documented LVEF measurements >52% within 12 months of the index date to improve specificity for preserved ejection fraction and reduce potential inclusion of HFmrEF or HFrEF patients. Outcome-specific denominators varied because patients with a documented history of the corresponding outcome prior to the beginning of each follow-up window were excluded from that specific analysis. PSM was performed without replacement using greedy nearest-neighbor matching with a caliper width of 0.1 pooled standard deviations of the logit of the propensity score. TriNetX analyses were conducted using available electronic health record data within the network. Missing laboratory or clinical variables were not imputed, and matching was performed using the covariate data available within the platform at the time of analysis. The TriNetX platform does not provide c-statistics or model discrimination metrics for the generated propensity score models.

## 3. Results

### 3.1. Patient Characteristics

A cohort of 30,254 patients with CAD and HFpEF who were treated with colchicine was identified, alongside 450,180 patients who did not receive colchicine. After propensity score matching (PSM), both groups were reduced to 28,941 each. Baseline characteristics before and after propensity score matching are reported in Supplemental Table S4. Before PSM, the mean age in the colchicine group was 71.2 ± 11.9 years, and in the no-colchicine, group, it was 72.2 ± 12.2 years (SMD = 0.077). White patients comprised 64.6% in the colchicine group versus 73.5% in the no-colchicine group (SMD = 0.193), while Black or African American patients accounted for 24.3% in the colchicine group compared with 16.4% in the no-colchicine group (SMD = 0.198). Females represented 43.1% of the colchicine cohort and 51.1% of the no-colchicine cohort (SMD = 0.160), whereas males constituted 56.8% and 48.9% of the colchicine and no-colchicine groups, respectively (SMD = 0.160). Regarding baseline comorbidities prior to matching, the prevalence in the colchicine group versus the no-colchicine group, respectively, was as follows: diabetes mellitus (62.7% vs. 46.1%; SMD = 0.339), chronic kidney disease (63.4% vs. 35.3%; SMD = 0.586), hypertensive diseases (96.7% vs. 82.6%; SMD = 0.474), hyperlipidemia (87.7% vs. 68.6%; SMD = 0.474), heart failure (97.7% vs. 59.9%; SMD = 1.045), acute kidney failure (57.7% vs. 28.5%; SMD = 0.618), atrial fibrillation (58.7% vs. 36.1%; SMD = 0.464), other cardiac arrhythmias (50.2% vs. 28.4%; SMD = 0.457), acute myocardial infarction (34.4% vs. 19.0%; SMD = 0.352), pulmonary heart disease and diseases of pulmonary circulation (43.2% vs. 22.6%; SMD = 0.448), overweight and obesity (58.5% vs. 33.2%; SMD = 0.526), and gout (54.2% vs. 9.0%; SMD = 1.112). Given the high baseline prevalence of gout in the colchicine cohort, residual confounding by indication may persist despite propensity score matching. For key medications, the colchicine group had 90.2% on beta blockers, 50.3% on ACE inhibitors/Angiotensin II inhibitors, 85.1% on statins, 90.2% on anticoagulants, and 86.1% on diuretics. In comparison, the non-colchicine group had 68.1%% on beta blockers, 34.05% on ACE inhibitors/Angiotensin II inhibitors, 63.4% on statins, and 52.1% on diuretics, indicating slight differences in medication use between the groups before matching.

After matching, the mean age in both cohorts was balanced at 71.5 ± 12.0 years (SMD = 0.004), comprising 43.25% women (SMD = 0.013), 65.5% White adults (SMD = 0.012), and 23.5% Black or African American adults (SMD = 0.011). Common comorbidities included hypertension (96.4%; SMD = 0.013), diabetes mellitus (61.9%; SMD = 0.008), chronic kidney diseases (62.5%; CKD = 0.005), obesity (57.1%; SMD = 0.009), hyperlipidemia (87.2%; SMD = 0.004), acute kidney failure (56.2%; SMD = 0.010), and atrial fibrillation (58.0%; SMD = 0.010). Additionally, 31.1% had old myocardial infarction (MI) (SMD = 0.006), while 32.9% (SMD = 0.012) had acute MI, and 52.6% (SMD = 0.013) had gout.

### 3.2. Primary Outcome

#### Primary Composite Outcome: AMI/ Stroke/All-Cause Mortality/Acute Heart Failure

**Figure 2:**
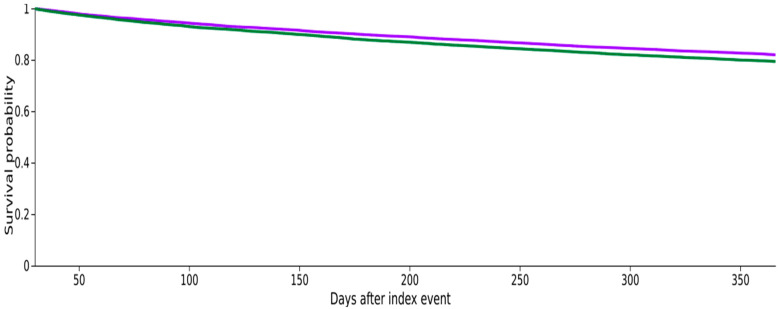
Kaplan–Meier curve for primary composite outcome: AMI/stroke/all-cause mortality/acute heart failure at 1-year follow-up.

At 1-year follow-up, the primary composite outcome (acute MI, stroke, all-cause mortality, or acute CHF) occurred at comparable rates between both groups. Primary composite events occurred in 2313 of 13,660 patients (16.9%) in the colchicine cohort and in 2577 of 12,998 patients (19.8%) in the non-colchicine cohort. Kaplan–Meier survival analysis revealed that colchicine use was associated with a statistically significant reduction in the hazard of the primary composite outcome during the 1-year follow-up period (HR: 0.86, 95% CI: 0.81 to 0.91; log-rank *p* < 0.001) (Table 1 and Figure 2).

**Figure 3:**
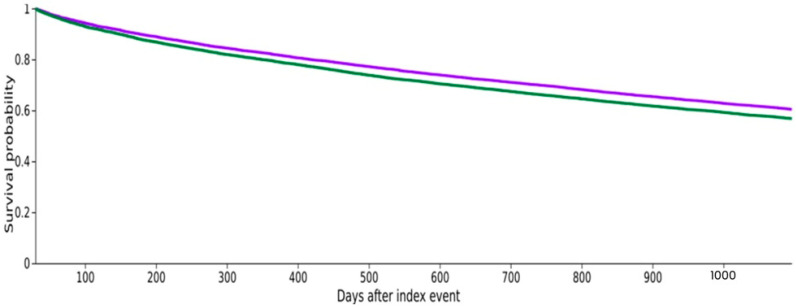
Kaplan–Meier curve for primary composite outcome: AMI/stroke/all-cause mortality/acute heart failure at 3-year follow-up.

During the 3-year follow-up period, the primary composite outcome (acute MI, stroke, all-cause mortality, or acute CHF) occurred in 4665 of 13,660 patients (34.2%) in the colchicine group compared with 5157 of 12,998 patients (39.7%) in the no-colchicine group. The Kaplan–Meier analysis showed that colchicine use was associated with a significantly lower risk of the primary composite outcome with an HR of 0.88 (95% CI: 0.85 to 0.92; log-rank *p* < 0.001) (Table 1 and Figure 3).

### 3.3. Secondary Outcomes

#### 3.3.1. All-Cause Mortality

At 1-year follow-up, all-cause mortality occurred in 3182 of 28,031 patients (11.4%) in the colchicine group, compared with 4008 of 27,713 patients (14.5%) in the non-colchicine group. Treatment with colchicine was associated with a statistically significant reduction in all-cause mortality compared to no colchicine use. Kaplan–Meier survival analysis demonstrated a hazard ratio (HR) of 0.77 (95% confidence interval [CI]: 0.73 to 0.80; log-rank *p* < 0.001) (Table 1 and Supplementary Figure S2).

At 3-year follow-up, all-cause mortality was observed in 6880 of 28,031 patients (24.5%) in the colchicine group, compared with 8207 of 27,213 patients (30.1%) in the no-colchicine group. Kaplan–Meier survival analysis showed an HR of 0.83 (95% CI: 0.80 to 0.86; log-rank *p* < 0.001), suggesting that colchicine was associated with a statistically significant risk reduction in the all-cause mortality outcome (Table 1 and Supplementary Figure S2).

#### 3.3.2. All-Cause Hospitalization

During the 1-year follow-up period, all-cause hospitalization occurred in 16,195 of 28,941 patients (55.9%) in the colchicine group and in 15,943 of 28,941 patients (55.0%) in the non-colchicine group. Kaplan–Meier survival analysis demonstrated a hazard ratio (HR) of 1.00 (95% CI: 0.98 to 1.02; log-rank *p* = 0.856), indicating no difference in the hazard of all-cause hospitalization between the groups, and this result was not statistically significant (Table 1 and Supplementary Figure S3).

Over a 3-year follow-up period, 20,251 patients (70.0%) in the colchicine group experienced all-cause hospitalization, compared to 19,916 patients (68.8%) in the matched non-colchicine group. Kaplan–Meier survival analysis showed an HR of 1.00 (95% CI: 0.99 to 1.02; log rank *p* = 0.381), suggesting no statistically significant association was observed between colchicine use and the risk of all-cause hospitalization compared to the no-colchicine group (Table 1 and Supplementary Figure S3).

#### 3.3.3. Stroke

At 1-year follow-up, stroke occurred in 1105 of 18,451 patients (6.0%) in the colchicine group compared with 1098 of 17,897 patients (6.1%) in the non-colchicine group. There was no statistically significant difference in stroke incidence between groups. Kaplan–Meier survival analysis demonstrated a HR of 0.96 (95% CI: 0.88 to 1.05; log-rank *p* = 0.351), confirming that colchicine use was not associated with a statistically significant reduction in stroke at 1 year (Table 1 and Supplementary Figure S4).

During the 3-year follow-up period, stroke occurred in 2307 of 18,451 patients (12.5%) in the colchicine group, whereas 2418 of 17,897 patients (13.5%) experienced stroke in the non-colchicine group. Kaplan–Meier survival analysis demonstrated an HR of 0.93 (95% CI: 0.88 to 0.99; log-rank *p* = 0.014), indicating a lower observed hazard of stroke in the colchicine group (Table 1 and Supplementary Figure S4).

#### 3.3.4. Gastrointestinal (GI) Symptoms

In the propensity score-matched analysis, during the 1-year follow-up period, 28,941 patients were included in each cohort. GI symptoms occurred in 8214 patients (28.4%) in the colchicine group and 8168 patients (28.2%) in the no-colchicine group. Kaplan–Meier analysis showed an HR of 0.98 (95% CI: 0.95 to 1.01; log-rank *p* = 0.230), suggesting a likely neutral effect of colchicine on GI symptoms risk (Table 1 and Supplementary Figure S5).

At 3-year follow-up, gastrointestinal (GI) symptoms occurred in 12,716 of 28,941 patients (43.9%) in the colchicine group and in 12,482 of 28,941 patients (43.1%) in the no-colchicine group. Kaplan–Meier analysis yielded a hazard ratio (HR) of 1.01 (95% CI: 0.99 to 1.04; log-rank *p* = 0.235), indicating no statistically significant difference in the risk of GI symptoms between the groups (Table 1 and Supplementary Figure S5).

#### 3.3.5. Acute Congestive Heart Failure

In the propensity score-matched cohort, 28,941 patients were included in each group. Acute congestive heart failure (CHF) occurred in 5216 patients (18.0%) in the colchicine group and in 5060 patients (17.5%) in the no-colchicine group. Kaplan–Meier analysis demonstrated an HR of 1.01 (95% CI: 0.98 to 1.05; log-rank *p* = 0.492). Overall, colchicine use was not associated with a significant increase in the risk of acute CHF in this matched population (Table 1 and Supplementary Figure S6).

During the 3-year follow-up period, acute CHF occurred in 7972 patients (27.5%) in the colchicine cohort compared with 7671 patients (26.5%) in the no-colchicine cohort. Kaplan–Meier analysis demonstrated that colchicine use was associated with a modest but statistically significant increased risk of acute CHF compared with no colchicine with an HR of 1.04 (95% CI: 1.01 to 1.08; log-rank *p* = 0.009). Overall, colchicine exposure was associated with a modest but statistically significant increase in the risk of acute CHF during follow-up (Table 1 and Supplementary Figure S6).

#### 3.3.6. Atrial Fibrillation (AF)

At 1-year follow-up, 11,710 patients in the colchicine group and 11,212 patients in the no-colchicine group were included. AF occurred in 910 patients (7.8%) in the colchicine group and in 841 patients (7.5%) in the no-colchicine group. Kaplan–Meier analysis showed an HR of 1.03 (95% CI: 0.94 to 1.13; log-rank *p* = 0.551), indicating no statistically significant association between colchicine use and AF (Table 1 and Supplementary Figure S7).

During 3-year follow-up, AF occurred in 1897 of 11,710 patients in the colchicine group (16.2%) and in 1813 of 11,212 patients in the non-colchicine group (16.2%). Kaplan–Meier survival analysis demonstrated no significant difference between the two groups (HR: 1.03; 95% CI: 0.96 to 1.09; log-rank *p* = 0.433), suggesting no statistically significant association between colchicine use and the risk of AF (Table 1 and Supplementary Figure S7).

### 3.4. Sensitivity Analysis Excluding Patients with Gout

Given the substantial baseline imbalance in gout prevalence prior to matching (54.2% in colchicine users versus 9.0% in non-users; SMD = 1.112), we performed a sensitivity analysis excluding patients with any documented diagnosis of gout (ICD-10 M10) prior to the index date. Following repeat 1:1 PSM using the same predefined covariates, the gout-excluded cohort included 2546 colchicine users and 2546 non-users. In this restricted cohort, the direction of association for the primary composite outcome remained generally consistent with the primary analysis, with HR values of 0.73 (95% CI: 0.58 to 0.92; log rank *p* = 0.007) at 1 year and 0.74 (95% CI: 0.63 to 0.87; log rank *p* < 0.001) at 3 years. Similarly, colchicine use remained associated with lower all-cause mortality at 1 year (HR: 0.56, 95% CI: 0.46 to 0.68). These findings suggest that the protective associations observed in the primary analysis were not solely attributable to the high baseline gout prevalence in colchicine users, although residual confounding by indication cannot be entirely excluded. Full sensitivity analysis results are presented in Supplemental Table S7.

### 3.5. Sensitivity Analysis Restricted to Patients with Documented Preserved LVEF

To address the potential misclassification associated with ICD-based identification of HFpEF, we performed a sensitivity analysis restricted to patients with at least one documented left ventricular ejection fraction (LVEF) measurement >52% within 12 months of the index date. An LVEF threshold >52% was selected to reduce overlap with borderline ejection fraction categories represented within grouped TriNetX reporting intervals.

After repeating 1:1 PSM using the same predefined covariates, the direction of association observed in the primary analysis remained generally consistent in the LVEF-confirmed cohort. The primary composite outcome remained consistent with the findings of the primary analysis, demonstrating a reduced risk in the colchicine group at both 1 year (HR: 0.73; 95% CI: 0.58 to 0.92; log-rank *p* = 0.007) and 3 years (HR: 0.74; 95% CI: 0.63 to 0.87; log-rank *p* < 0.001). In addition, colchicine therapy continued to be associated with a lower risk of all-cause mortality at 1 year (HR: 0.56; 95% CI: 0.46 to 0.68) (Supplemental Table S7).

## 4. Discussion

**Figure 4:**
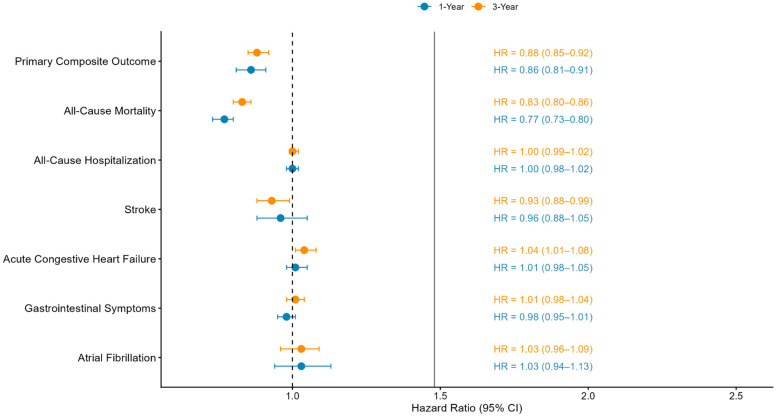
Forest plot for one-year and three-year follow-up clinical outcomes in patients with heart failure with preserved ejection fraction (HFpEF) and coronary artery disease treated with colchicine versus no colchicine after propensity score matching.

In this large propensity-matched cohort of patients with CAD and HFpEF, colchicine was associated with a lower observed rate in all-cause mortality and the composite cardiovascular outcome at 1 year. Although the association persisted for 3 years, the magnitude of risk reduction was attenuated over time. For stroke, colchicine was not associated with a reduction at 1 year, but a modest, statistically significant reduction was observed at 3 years, suggesting a potential time-dependent effect. Colchicine was not associated with reductions in hospitalization or atrial fibrillation, and a small increase in acute heart failure events emerged during longer-term follow-up (Figure 4). Importantly, the observed increase in acute heart failure events at 3 years should be interpreted in the context of competing risk dynamics and differential survival. Patients in the colchicine group experienced a lower all-cause mortality rate over the follow-up period, which may have increased the number of individuals at risk of subsequently developing and being coded for heart failure events. In contrast, higher early mortality in the comparator group may have precluded the occurrence or capture of later heart failure diagnoses. Therefore, the observed association may partly reflect a “survivor-to-be-hospitalized” phenomenon rather than a true pro-hypertensive or cardiotoxic effect of colchicine. Overall, these findings demonstrate a temporal pattern in the observed associations between colchicine exposure and cardiovascular outcomes in CAD-HFpEF. However, because colchicine exposure was modeled as a time-fixed rather than time-varying variable, these temporal patterns should be interpreted cautiously, as changes in medication use during follow-up could not be fully accounted for.

The early associations with lower mortality observed in our study may be consistent with previously described inflammatory mechanisms in HFpEF pathophysiology [10]. However, the present analysis did not include circulating inflammatory biomarkers, imaging data, or functional mechanistic measures; therefore, biological interpretations remain speculative. Prior experimental work has suggested potential involvement of inflammatory pathways in CAD and HFpEF [11,12], and colchicine has been hypothesized to modulate cardiovascular risk through inhibition of the NLRP3 inflammasome and downstream interleukin-1β signaling [13,14]. Accordingly, these pathways may provide a biological framework for the observed associations rather than confirmatory mechanistic evidence. This interpretation is supported by recent pilot data (Shi et al., 2026) [15], which reported reductions in circulating inflammatory cytokines and possible improvements in clinical symptoms in HFpEF populations, although these findings remain preliminary. Furthermore, preclinical (rat) models suggest that colchicine can alleviate myocardial inflammation and improve diastolic dysfunction [16], providing a potential mechanistic hypothesis for the 1-year mortality associations observed in our study. Importantly, HFpEF is also characterized by coronary microvascular rarefaction and progressive myocardial fibrosis, which contribute to impaired ventricular compliance and long-term disease progression [17], offering additional context for the attenuation of observed associations over time.

Prior randomized controlled trials, including the Colchicine Cardiovascular Outcomes Trial (COLCOT) and LoDoCo2, have demonstrated reductions in cardiovascular events with colchicine therapy among selected patients with coronary artery disease [18,19]. However, these trials differ substantially from the present study in terms of study design, inclusion criteria, baseline risk profiles, and clinical setting; therefore, direct comparisons with the current observational cohort of patients with coexisting CAD and HFpEF should be made cautiously. In contrast, randomized trials conducted in acute coronary syndrome populations, including the COPS and CLEAR SYNERGY trials, have reported more heterogeneous and less consistent results [20,21]. Importantly, none of these trials specifically enrolled patients with HFpEF, leaving a key evidence gap regarding this combined phenotype. Our study adds to existing evidence regarding these observations to a high-risk cohort with diastolic dysfunction, myocardial fibrosis, and substantial comorbidity burden, and the associations observed should be interpreted cautiously, as these characteristics may limit the long-term efficacy of anti-inflammatory therapy alone. Importantly, the magnitude of association observed for all-cause mortality was greater than that observed for individual cardiovascular endpoints such as stroke, while AMI-specific analyses were limited by TriNetX de-identification thresholds. This discrepancy warrants cautious interpretation because all-cause mortality in observational pharmaco-epidemiologic studies is particularly susceptible to healthy-user bias, healthy-adherer bias, and residual frailty-related confounding. Patients prescribed colchicine may differ systematically from non-users in ways not fully captured through electronic health record data, including healthcare engagement, medication adherence, clinician follow-up intensity, or baseline functional status. Although propensity score matching balanced 67 measured covariates, including major cardiovascular therapies such as statins, beta blockers, ACE inhibitors/ARBs, anticoagulants, and SGLT2 inhibitors, unmeasured confounding cannot be excluded. Accordingly, the observed mortality association should be interpreted cautiously and considered hypothesis-generating rather than definitive evidence of a causal survival benefit.

### 4.1. Future Directions

Future prospective studies are needed to clarify the role of colchicine in patients with coexisting CAD and HFpEF. Randomized controlled trials targeting this phenotype are required to determine whether the observed associations reflect true disease modification or transient effects related to inflammation. HFpEF is a heterogeneous syndrome in which multiple mechanisms, including inflammation, endothelial dysfunction, and myocardial remodeling, have been proposed [11,12]; however, these pathways could not be assessed in the present study. Future investigations incorporating inflammatory biomarkers, advanced imaging, and echocardiographic characterization may help better define patient subgroups. The potential influence of dose, duration, and adherence on the observed temporal patterns also warrants further study.

### 4.2. Limitations

Our study has several limitations. First, because of its observational design, causality cannot be established despite the use of proper propensity score matching. Second, HFpEF identification using ICD codes without echocardiographic confirmation of preserved ejection fraction may have led to misclassification of HFmrEF or HFrEF.

Third, the information regarding different colchicine doses, durations, and adherence levels was not consistently available, limiting evaluation of exposure intensity and time-dependent effects. Although we performed a sensitivity analysis excluding patients with gout, residual confounding by indication may still persist because colchicine dose, indication, adherence, and treatment duration were unavailable within the TriNetX platform. Exposure was treated as time-fixed, and changes in colchicine use over time (e.g., discontinuation or delayed initiation) could not be captured.

Fourth, important variables relevant for HFpEF severity, such as echocardiographic parameters and biomarker data, were unavailable, which may affect both prognosis and treatment selection. Additionally, HFpEF was identified using ICD codes alone without echocardiographic confirmation of preserved ejection fraction, which may have led to misclassification, potentially including patients with reduced or mildly reduced ejection fraction. Although HFpEF was initially identified using ICD-based diagnostic coding, which may introduce misclassification between HFpEF, HFmrEF, and HFrEF, we performed an additional sensitivity analysis restricted to patients with documented preserved LVEF (>52%), and the principal associations remained directionally consistent. Nevertheless, echocardiographic parameters, diastolic function grading, and comprehensive imaging data were not uniformly available within TriNetX, and some residual phenotype misclassification may persist. Additionally, cardiovascular mortality could not be analyzed separately because TriNetX does not provide consistently adjudicated cause-specific mortality data across participating healthcare organizations. Independent AMI analyses were also limited because portions of the matched outcome data were suppressed by TriNetX de-identification thresholds for low event counts, preventing stable estimation of AMI-specific hazard ratios. Furthermore, the dominant association observed for all-cause mortality relative to more specific cardiovascular endpoints raises the possibility of residual healthy-user bias, healthy-adherer bias, and unmeasured frailty-related confounding despite extensive propensity score matching.

Despite propensity score matching, residual confounding may persist, particularly for gout, which was substantially more prevalent in the colchicine group and could influence outcomes; this likely reflects confounding by indication, and subgroup or sensitivity analyses were not possible. Gastrointestinal outcomes were defined broadly using ICD codes R10–R19, capturing nonspecific symptoms that may not reflect true colchicine-related adverse effects. Furthermore, competing risk methodologies could not be applied in this study due to the limitations of the TriNetX platform, which does not support Fine–Gray subdistribution hazard modeling. As a result, acute heart failure events were analyzed using standard Cox proportional hazards models without explicitly accounting for the competing risk of death. Given the significantly lower all-cause mortality observed in the colchicine group, the modest increase in acute heart failure at 3 years may reflect differential survival rather than a direct adverse effect. In other words, patients receiving colchicine were more likely to survive long enough to manifest or be diagnosed with heart failure events, whereas higher mortality in the comparator group may have reduced the observed incidence of subsequent heart failure outcomes. This potential survival-related detection bias should be considered when interpreting the long-term heart failure signal.

## 5. Conclusions

In this propensity-matched observational cohort of patients with lower observed rates of all-cause mortality and composite cardiovascular events, these findings remained generally consistent in sensitivity analyses restricted to patients with documented preserved LVEF, although residual confounding and phenotype misclassification cannot be excluded. Accordingly, these findings should be considered hypothesis-generating and warrant prospective validation in rigorously phenotyped HFpEF populations.

## Figures and Tables

**Table 1:** One- and Three-Year Follow-up Clinical Outcomes in Patients With Heart Failure with Preserved Ejection Fraction (HFpEF) and Coronary Artery Disease Treated With Colchicine Versus No Colchicine After Propensity Score Matching.

Outcome	1-Year Colchicine (n/N)	1-Year No Colchicine (n/N)	HR (95% CI)	Log-Rank *p* Value	3-Year Colchicine (n/N)	3-Year No Colchicine (n/N)	HR (95% CI)	Log-Rank *p* Value
Primary Composite Outcome (AMI/Stroke/All-Cause Mortality/Acute HF)	2313/13,660	2577/172,998	0.86 (0.81–0.91)	<0.001	4665/13,660	5157/12,998	0.88 (0.85–0.92)	<0.001
All-Cause Mortality	3182/28,031	4008/27,713	0.77 (0.73–0.80)	<0.001	6880/28,031	8207/27,213	0.83 (0.80–0.86)	<0.001
All-Cause Hospitalization	16,195/28,941	15,943/28,941	1.00 (0.98–1.02)	0.856	20,251/28,941	19,916/28,941	1.00 (0.99–1.02)	0.381
Stroke	1105/18,451	1098/17,897	0.96 (0.88–1.05)	0.351	2307/18,451	2418/17,897	0.93 (0.88–0.99)	0.014
Acute Congestive Heart Failure	5216/28,941	5060/28,941	1.01 (0.98–1.05)	0.492	7972/28,941	7671/28,941	1.04 (1.01–1.08)	0.009
Gastrointestinal Symptoms	8214/28,941	8168/28,941	0.98 (0.95–1.01)	0.23	12,716/28,941	12,482/28,941	1.01 (0.99–1.04)	0.235
Atrial Fibrillation	910/11,710	841/11,212	1.03 (0.94–1.13)	0.551	1897/11,710	1813/11,212	1.03 (0.96–1.09)	0.433

HR = Hazard Ratio; CI = Confidence Interval. n= population with outcome, N = total population of cohort.

## Data Availability

The data that support the findings of this study are available from the TriNetX Research Network. Due to licensing restrictions and data use agreements, the raw data are not publicly available. Access to the TriNetX platform can be obtained through institutional subscription. Aggregate data supporting the findings of this study may be available from the corresponding author upon reasonable request and with permission from TriNetX.

## Supplementary Materials

The following supporting information can be downloaded at: https://www.mdpi.com/article/10.3390/jcdd13060222/s1, Table S1: Query criteria for Cohort 1 (query name: colchicine); Table S2: Query criteria for Cohort 2 (query name: no colchicine); Table S3: Outcome definitions; Table S4: Baseline demographic and clinical characteristics before and after propensity score matching; Table S5: ICD-9 and ICD-10 diagnosis codes used to define cardiovascular population, comorbidities and outcomes; Table S6: Full propensity score model specification constructed from both the 1-year and 3-year analyses; Table S7: Sensitivity analysis excluding gout with three-year and one-year outcomes in patients with HFpEF and CAD: colchicine vs no colchicine (propensity score-matched cohorts); Table S8: Outcome-specific denominators, prior event exclusions, and event counts in the propensity-matched cohort; Figure S1: Propensity score density function: before and after matching; Figure S2: Kaplan–Meier curves for all-cause mortality for 1-year and 3-year follow-up respectively; Figure S3: Kaplan–Meier curves for all-cause hospitalization for 1-year and 3-year follow-up respectively; Figure S4: Kaplan–Meier curves for stroke for 1-year and 3-year follow-up respectively; Figure S5: Kaplan–Meier curves for GI symptoms for 1-year and 3-year follow-up respectively; Figure S6: Kaplan–Meier curves for acute CHF for 1-year and 3-year follow-up respectively; Figure S7: Kaplan–Meier Curves for atrial fibrillation for 1-year and 3-year follow-up respectively.
