# Supplementary material for: Cardiovascular Outcomes with Colchicine in Coronary Artery Disease and HFpEF: A Propensity-Matched TriNetX Analysis
Source: J Cardiovasc Dev Dis. 2026 May 23;13(6):222. doi: 10.3390/jcdd13060222 (PMC13301838; doi:10.3390/jcdd13060222)
Supplement: Supplementary file 1 [file jcdd-13-00222-s001.zip › jcdd-4275191-supplementary.pdf]

Supplemental Table S1: Query criteria for Cohort 1 (query name: colchicine).

This query was run on the network, with 107 HCO(s) queried and 107 HCO(s) responded. A total of 66 provider(s) responded with patients. The final cohort included 30,254 patients who matched the query criteria listed in the table below. For the text representation of the query criteria please see Appendix A.

**Ungrouped terms**				
must have		demographics	Age	Age (at least 18 years (most recent occurrence))
**Group 1**				
**Group 1A**				
must have	any of	diagnosis	UMLS:ICD9CM:428.3	Diastolic heart failure
		diagnosis	UMLS:ICD10CM:I50.32	Chronic diastolic (congestive) heart failure
		diagnosis	UMLS:ICD9CM:414	Other forms of chronic ischemic heart disease
		diagnosis	UMLS:ICD10CM:I50.30	Unspecified diastolic (congestive) heart failure
	and any of	diagnosis	UMLS:ICD9CM:414.0	Coronary atherosclerosis
		diagnosis	UMLS:ICD10CM:I21	Acute myocardial infarction
		diagnosis	UMLS:ICD10CM:I25.8	Other forms of chronic ischemic heart disease
		diagnosis	UMLS:ICD10CM:I24	Other acute ischemic heart diseases
		diagnosis	UMLS:ICD10CM:I25.2	Old myocardial infarction
		diagnosis	UMLS:ICD10CM:I25.1	Atherosclerotic heart disease of native coronary artery
		diagnosis	UMLS:ICD10CM:I25.7	Atherosclerosis of coronary artery bypass graft(s) and coronary artery of transplanted heart with angina pectoris
		diagnosis	UMLS:ICD10CM:I22	Subsequent ST elevation (STEMI) and non-ST elevation (NSTEMI) myocardial infarction
		diagnosis	UMLS:ICD10CM:I20	Angina pectoris
		diagnosis	UMLS:ICD10CM:I23	Certain current complications following ST elevation (STEMI) and non-ST elevation (NSTEMI) myocardial infarction (within the 28 day period)
		diagnosis	UMLS:ICD9CM:411	Other acute and subacute forms of ischemic heart disease
		diagnosis	UMLS:ICD10CM:I25.6	Silent myocardial ischemia
		diagnosis	UMLS:ICD10CM:I25.9	Chronic ischemic heart disease, unspecified
date constraint		This group occurred before 3 years ago		
event relationship		Any instance of Group 1B occurred on or after any instance of Group 1A		
**Group 1B**				
must have		medication	NLM:RXNORM:2683	colchicine

Supplemental Table S2: Query criteria for Cohort 2 (query name: no colchicine).

This query was run on the network, with 107 HCO(s) queried and 107 HCO(s) responded. A total of 73 provider(s) responded with patients. The final cohort included 450,180 patients who matched the query criteria listed in the table below.

**Ungrouped terms**				
must have		demographics	Age	Age (at least 18 years (most recent occurrence))
**Group 1**				
**Group 1A**				
must have	any of	diagnosis	UMLS:ICD9CM:428.3	Diastolic heart failure
		diagnosis	UMLS:ICD10CM:I50.32	Chronic diastolic (congestive) heart failure
		diagnosis	UMLS:ICD9CM:414	Other forms of chronic ischemic heart disease
		diagnosis	UMLS:ICD10CM:I50.30	Unspecified diastolic (congestive) heart failure
	and any of	diagnosis	UMLS:ICD9CM:414.0	Coronary atherosclerosis
		diagnosis	UMLS:ICD10CM:I21	Acute myocardial infarction
		diagnosis	UMLS:ICD10CM:I25.8	Other forms of chronic ischemic heart disease
		diagnosis	UMLS:ICD10CM:I24	Other acute ischemic heart diseases
		diagnosis	UMLS:ICD10CM:I25.2	Old myocardial infarction
		diagnosis	UMLS:ICD10CM:I25.1	Atherosclerotic heart disease of native coronary artery
		diagnosis	UMLS:ICD10CM:I25.7	Atherosclerosis of coronary artery bypass graft(s) and coronary artery of transplanted heart with angina pectoris
		diagnosis	UMLS:ICD10CM:I22	Subsequent ST elevation (STEMI) and non-ST elevation (NSTEMI) myocardial infarction
		diagnosis	UMLS:ICD10CM:I20	Angina pectoris
		diagnosis	UMLS:ICD10CM:I23	Certain current complications following ST elevation (STEMI) and non-ST elevation (NSTEMI) myocardial infarction (within the 28 day period)
		diagnosis	UMLS:ICD9CM:411	Other acute and subacute forms of ischemic heart disease

	diagnosis	UMLS:ICD10CM:I25.6	Silent myocardial ischemia
	diagnosis	UMLS:ICD10CM:I25.9	Chronic ischemic heart disease, unspecified
date constraint	This group occurred before 3 years ago		
event relationship	Any instance of Group 1B occurred on or after any instance of Group 1A		
**Group 1B**			
cannot have	medication	NLM:RXNORM:2683	colchicine

Supplemental Table S3: Outcome definitions.

The table below outlines the definitions for each outcome and the analysis specifications. For outcome definitions consisting of more than one term, at least one term must match. Please see Appendix C for the text representation of the outcome definitions.

**All-cause mortality**		
**Outcome definition**		
Demographics	Deceased	Deceased
**Settings for the performed analyses**		
Risk analysis		Excluding patients with an outcome prior to the time window
Kaplan–Meier survival analysis		Excluding patients with an outcome prior to the time window
Number of instances analysis		Excluding patients with an outcome prior to the time window<br>Including patients with zero outcomes<br>Counts are grouped by date
**All-cause Hospitalization/ER Visits**		
**Outcome definition**		
Visit	UMLS:HL7V3.0:VisitType:EMER	Visit: Emergency
Visit	UMLS:HL7V3.0:VisitType:ACUTE	Visit: Inpatient Acute
Visit	UMLS:HL7V3.0:VisitType:IMP	Visit: Inpatient Encounter
Visit	UMLS:HL7V3.0:VisitType:NONAC	Visit: Inpatient Non-acute
Visit	UMLS:HL7V3.0:VisitType:OBSENC	Visit: Observation Encounter
Visit	UMLS:HL7V3.0:VisitType:SS	Visit: Short Stay
**Settings for the performed analyses**		
Risk analysis		Including patients with an outcome prior to the time window

Kaplan–Meier survival analysis		Including patients with an outcome prior to the time window
Number of instances analysis		Including patients with an outcome prior to the time window<br>Including patients with zero outcomes<br>Counts are grouped by date
**Stroke/CVA**		
**Outcome definition**		
Diagnosis	UMLS:ICD10CM:I60-I69	Cerebrovascular diseases
Diagnosis	UMLS:ICD10CM:I63	Cerebral infarction
**Settings for the performed analyses**		
Number of instances analysis		Excluding patients with an outcome prior to the time window<br>Including patients with zero outcomes<br>Counts are grouped by date
Kaplan–Meier survival analysis		Excluding patients with an outcome prior to the time window
Risk analysis		Excluding patients with an outcome prior to the time window
**Primary Composite Outcome: AMI/Stroke/All-cause mortality/Acute Heart Failure**		
**Outcome definition**		
Demographics	Deceased	Deceased
Diagnosis	UMLS:ICD10CM:I63	Cerebral infarction
Diagnosis	UMLS:ICD10CM:I50.31	Acute diastolic (congestive) heart failure
Diagnosis	UMLS:ICD10CM:I50.33	Acute on chronic diastolic (congestive) heart failure
Diagnosis	UMLS:ICD10CM:I22	Subsequent ST elevation (STEMI) and non-ST elevation (NSTEMI) myocardial infarction
**Settings for the performed analyses**		
Risk analysis		Excluding patients with an outcome prior to the time window
Kaplan - Meier survival analysis		Excluding patients with an outcome prior to the time window
Number of instances analysis		Excluding patients with an outcome prior to the time window<br>Including patients with zero outcomes<br>Counts are grouped by date
**GI Symptoms**		
**Outcome definition**		
Diagnosis	UMLS:ICD10CM:R10	Abdominal and pelvic pain
Diagnosis	UMLS:ICD10CM:R11	Nausea and vomiting
Diagnosis	UMLS:ICD10CM:R14	Flatulence and related conditions
Diagnosis	UMLS:ICD10CM:R15	Fecal incontinence
Diagnosis	UMLS:ICD10CM:R17	Unspecified jaundice
Diagnosis	UMLS:ICD10CM:R19.0	Intra-abdominal and pelvic swelling, mass and lump
Diagnosis	UMLS:ICD10CM:R19.1	Abnormal bowel sounds
Diagnosis	UMLS:ICD10CM:R19.2	Visible peristalsis
Diagnosis	UMLS:ICD10CM:R19.3	Abdominal rigidity
Diagnosis	UMLS:ICD10CM:R19.4	Change in bowel habit
Diagnosis	UMLS:ICD10CM:R19.5	Other fecal abnormalities

Diagnosis	UMLS:ICD10CM:R19.6	Halitosis
Diagnosis	UMLS:ICD10CM:R19.7	Diarrhea, unspecified
Diagnosis	UMLS:ICD10CM:R19.8	Other specified symptoms and signs involving the digestive system and abdomen
**Settings for the performed analyses**		
Risk analysis		Including patients with an outcome prior to the time window
Kaplan–Meier survival analysis		Including patients with an outcome prior to the time window
Number of instances analysis		Including patients with an outcome prior to the time window<br>Including patients with zero outcomes<br>Counts are grouped by date
**Acute CHF**		
**Outcome definition**		
Diagnosis	UMLS:ICD10CM:I50.31	Acute diastolic (congestive) heart failure
Diagnosis	UMLS:ICD10CM:I50.33	Acute on chronic diastolic (congestive) heart failure
**Settings for the performed analyses**		
Risk analysis		Including patients with an outcome prior to the time window
Kaplan–Meier survival analysis		Including patients with an outcome prior to the time window
Number of instances analysis		Including patients with an outcome prior to the time window<br>Including patients with zero outcomes<br>Counts are grouped by date
**Atrial Fibrillation**		
**Outcome definition**		
Diagnosis	UMLS:ICD10CM:I48	Atrial fibrillation and flutter
**Settings for the performed analyses**		
Risk analysis		Excluding patients with an outcome prior to the time window
Kaplan–Meier survival analysis		Excluding patients with an outcome prior to the time window
Number of instances analysis		Including patients with an outcome prior to the time window<br>Including patients with zero outcomes<br>Counts are grouped by date

Supplemental Table S4. Baseline demographic and clinical characteristics before and after propensity score matching.

Characteristic	Colchicine Group (N = 30,254)	No-Colchicine Group (N = 450,180)	Std Diff.	Colchicine Group (N = 28,941)	No-Colchicine Group (N = 28,941)	Std Diff.

	Before PSM			After PSM		
Demographics						
**Age, years (mean ± SD)**	71.2 ± 11.9	72.2 ± 12.2	0.077	71.4 ± 11.9	71.4 ± 12.2	0.004
**White**	19,515 (64.6%)	329,354 (73.5%)	0.193	18,870 (65.2%)	19,029 (65.8%)	0.012
**Black/African American**	7,339 (24.3%)	73,349 (16.4%)	0.198	6,894 (23.8%)	6,753 (23.3%)	0.011
**Asian**	1,154 (3.8%)	12,594 (2.8%)	0.056	1,106 (3.8%)	1,110 (3.8%)	0.001
**Female**	13,017 (43.1%)	228,806 (51.1%)	0.160	12,610 (43.6%)	12,418 (42.9%)	0.013
**Male**	17,160 (56.8%)	218,847 (48.9%)	0.160	16,322 (56.4%)	16,503 (57.0%)	0.013
Comorbidities						
**Diabetes mellitus**	18,923 (62.7%)	206,266 (46.1%)	0.339	17,967 (62.1%)	17,855 (61.7%)	0.008
**Chronic kidney disease**	19,140 (63.4%)	158,058 (35.3%)	0.586	18,063 (62.4%)	18,133 (62.7%)	0.005
**Hypertensive diseases**	29,182 (96.7%)	370,042 (82.6%)	0.474	27,942 (96.5%)	27,873 (96.3%)	0.013
**Overweight/Obesity**	17,660 (58.5%)	148,574 (33.2%)	0.526	16,591 (57.3%)	16,456 (56.9%)	0.009
**Hyperlipidemia**	26,463 (87.7%)	307,194 (68.6%)	0.474	25,256 (87.3%)	25,217 (87.1%)	0.004
**Atrial fibrillation/flutter**	17,709 (58.7%)	161,719 (36.1%)	0.464	16,725 (57.8%)	16,866 (58.3%)	0.01
**Heart failure**	29,503 (97.7%)	268,132 (59.9%)	1.045	28,257 (97.6%)	28,166 (97.3%)	0.02
**Acute myocardial infarction**	10,370 (34.4%)	85,256 (19.0%)	0.352	9,621 (33.2%)	9,451 (32.7%)	0.012
**Gout**	16,362 (54.2%)	40,332 (9.0%)	1.112	15,135 (52.3%)	15,329 (53.0%)	0.013
Medications						
**Digitalis glycosides**	3,980 (13.2%)	29,170 (6.5%)	0.225	3,698 (12.8%)	3,784 (13.1%)	0.009
**Beta blockers**	27,242 (90.2%)	305,208 (68.1%)	0.566	26,018 (89.9%)	26,008 (89.9%)	0.001
**Calcium channel blockers**	21,560 (71.4%)	220,428 (49.2%)	0.466	20,490 (70.8%)	20,518 (70.9%)	0.002

**Antiarrhythmics**	24,366 (80.7%)	248,112 (55.4%)	0.564	23,175 (80.1%)	23,206 (80.2%)	0.003
**Antilipemic agents**	25,682 (85.1%)	283,995 (63.4%)	0.511	24,482 (84.6%)	24,493 (84.6%)	0.001
**ACE inhibitors**	17,140 (56.8%)	182,977 (40.9%)	0.323	16,303 (56.3%)	16,156 (55.8%)	0.01
**Angiotensin II inhibitors**	13,249 (43.9%)	121,760 (27.2%)	0.354	12,416 (42.9%)	12,561 (43.4%)	0.01
**Sacubitril**	1,653 (5.5%)	8,181 (1.8%)	0.195	1,422 (4.9%)	1,467 (5.1%)	0.007
**Thiazides/related diuretics**	15,225 (50.4%)	134,095 (29.9%)	0.427	14,274 (49.3%)	14,155 (48.9%)	0.008
**Loop diuretics**	26,463 (87.7%)	263,965 (58.9%)	0.686	25,249 (87.2%)	25,173 (87.0%)	0.008
**Potassium-sparing/combination diuretics**	10,280 (34.1%)	69,071 (15.4%)	0.442	9,430 (32.6%)	9,391 (32.4%)	0.003
**Hydralazine**	13,081 (43.3%)	117,265 (26.2%)	0.366	12,330 (42.6%)	12,276 (42.4%)	0.004
**Nitroglycerin**	16,150 (53.5%)	136,759 (30.5%)	0.478	15,133 (52.3%)	15,118 (52.2%)	0.001
**Insulin**	17,637 (58.4%)	167,840 (37.5%)	0.429	16,725 (57.8%)	16,728 (57.8%)	<0.001
**Oral hypoglycemic agents**	12,330 (40.8%)	110,513 (24.7%)	0.350	11,472 (39.6%)	11,493 (39.7%)	0.001
**GLP-1 analogs**	2,628 (8.7%)	15,412 (3.4%)	0.222	2,291 (7.9%)	2,251 (7.8%)	0.005
**SGLT2 inhibitors**	3,189 (10.6%)	13,976 (3.1%)	0.298	2,631 (9.1%)	2,589 (8.9%)	0.005
**Anticoagulants**	27,226 (90.2%)	292,606 (65.3%)	0.626	25,989 (89.8%)	25,964 (89.7%)	0.003
**NSAIDs**	18,596 (61.6%)	167,011 (37.3%)	0.501	17,526 (60.6%)	17,386 (60.1%)	0.01
**Laboratory Data**						
**LVEF (%)**	54.2 ± 14.6	55.6 ± 14.1	0.098	54.4 ± 14.5	52.9 ± 15.4	0.1
**Hemoglobin A1c (%)**	6.5 ± 1.6	6.6 ± 1.7	0.04	6.5 ± 1.6	6.5 ± 1.6	<0.001
**BNP (pg/mL)**	803 ± 2484.1	794 ± 2593	0.004	805.3 ± 2472.8	803.6 ± 2537.6	0.001

**Glomerular Filtration Rate (mL/min/1.73m^2)**	52.7 ± 26.8	60.1 ± 30.1	0.260	52.9 ± 27.0	52.5 ± 28.7	0.013
**INR**	1.4 ± 0.7	1.3 ± 0.7	0.143	1.4 ± 0.7	1.4 ± 0.7	0.006

Supplemental Table S5. ICD-9 and ICD-10 diagnosis codes used to define cardiovascular population, comorbidities and outcomes.

Disease / Condition	ICD-9 Code(s)	ICD-10 Code(s)
**Coronary Artery Disease (CAD)**	414, 414.0, 411	I20, I21, I22, I23, I24, I25.1, I25.2, I25.5, I25.6, I25.7, I25.8, I25.9
**Heart Failure with Preserved Ejection Fraction (HFpEF)**	428.3	I50.30, I50.32
Acute Diastolic Heart Failure	-	I50.31
Acute on Chronic Diastolic Heart Failure	-	I50.33
Heart Failure (General)	-	I50
Acute Myocardial Infarction (AMI)	-	I21
Subsequent STEMI/NSTEMI	-	I22
Old Myocardial Infarction	-	I25.2
Post-MI Complications (≤28 days)	-	I23
Other Acute Ischemic Heart Disease	411	I24
Silent Myocardial Ischemia	-	I25.6
Angina Pectoris (All types)	-	I20, I20.0, I20.8, I20.9
Chronic Ischemic Heart Disease	414	I25.8, I25.9
Atherosclerotic Heart Disease (Native Coronary)	-	I25.1
Atherosclerosis of CABG/Transplanted Heart	-	I25.7
Cerebrovascular Disease (Stroke/CVA)	-	I60–I69
Cerebral Infarction	-	I63
Atrial Fibrillation and Flutter	-	I48
Other Cardiac Arrhythmias	-	I49

Atrioventricular and Left Bundle Branch Block	-	I44
Other Conduction Disorders	-	I45
Atherosclerosis (Peripheral)	-	I70
Diseases of Arteries, Arterioles, Capillaries	-	I70–I79
Hypertensive Diseases	-	I10–I1A
Ischemic Cardiomyopathy	-	I25.5
Cardiomyopathy (Elsewhere Classified)	-	I43
Pulmonary Heart Disease	-	I26–I28
Acute Pericarditis	-	I30
Diabetes Mellitus	-	E08–E13
Chronic Kidney Disease (CKD)	-	N18
Acute Kidney Failure	-	N17
Overweight and Obesity	-	E66
Hypothyroidism (Other)	-	E03
Disorders of Lipid Metabolism	-	E78
Gout	-	M10
Systemic Lupus Erythematosus (SLE)	-	M32
Rheumatoid Arthritis (Unspecified)	-	M06.9
Rheumatoid Arthritis and Polyarthropathies	714	-
Fibrosis and Cirrhosis of Liver	-	K74
Chronic Lower Respiratory Diseases	-	J40–J4A
Tobacco Use	-	Z72.0
External Causes of Morbidity	-	V00–Y99
Presence of Aortocoronary Bypass Graft	-	Z95.1
Presence of Cardiac Pacemaker	-	Z95.0
Personal History of TIA/Stroke	-	Z86.73
Abdominal and Pelvic Pain	-	R10
Nausea and Vomiting	-	R11
Flatulence	-	R14
Fecal Incontinence	-	R15

Unspecified Jaundice	-	R17
Abdominal/Pelvic Mass	-	R19.0
Abnormal Bowel Sounds	-	R19.1
Visible Peristalsis	-	R19.2
Abdominal Rigidity	-	R19.3
Change in Bowel Habit	-	R19.4
Other Fecal Abnormalities	-	R19.5
Halitosis	-	R19.6
Diarrhea (Unspecified)	-	R19.7
Other Digestive Symptoms	-	R19.8

Supplemental Table S6: Full propensity score model specification constructed from both the 1-year and 3-year analyses.

Parameter	1-Year Follow-up Analysis	3-Year Follow-up Analysis
**Model type**	Logistic regression (propensity score model generated within the TriNetX platform)	Logistic regression (propensity score model generated within the TriNetX platform)
**All variables included in the PS model**	Age, sex, race, hypertension, diabetes mellitus, chronic kidney disease, atrial fibrillation, prior myocardial infarction, prior stroke, obesity, hyperlipidemia, smoking history, baseline medications (antiplatelets, beta-blockers, ACE inhibitors/ARBs, statins, diuretics), and other baseline cardiovascular comorbidities	Age, sex, race, hypertension, diabetes mellitus, chronic kidney disease, atrial fibrillation, prior myocardial infarction, prior stroke, obesity, hyperlipidemia, smoking history, baseline medications (antiplatelets, beta-blockers, ACE inhibitors/ARBs, statins, diuretics), and other baseline cardiovascular comorbidities
**Matching ratio**	1:1 nearest-neighbor matching	1:1 nearest-neighbor matching
**Caliper width**	0.1 pooled standard deviations	0.1 pooled standard deviations

**% matched**	100% of a smaller cohort matched	100% of a smaller cohort matched
**Number excluded after matching**	Patients unmatched from a larger cohort (difference between pre- and post-match sample sizes)	Patients unmatched from a larger cohort (difference between pre- and post-match sample sizes)

Supplemental Table S7. Sensitivity analysis excluding gout with three-year and one-year outcomes in patients with HFpEF and CAD: colchicine vs no colchicine (propensity score-matched cohorts).

Outcome	1-Year Colchicine (n/N)	1-Year No Colchicine (n/N)	HR (95% CI)	Log-rank p Value	3-Year Colchicine (n/N)	3-Year No Colchicine (n/N)	HR (95% CI)	Log-rank p Value
**Primary Outcome**								
**Primary Composite Outcome (AMI/Stroke/All-Cause Mortality/Acute HF)**	**121 / 1,056**	**173 / 1,096**	**0.73 (0.58–0.92)**	**0.007**	**247/1,056**	**364/1,096**	**0.74 (0.63-0.87)**	**<0.001**
**Secondary Outcomes**								

**All-Cause Mortality**	**160 / 2,489**	**281 / 2,455**	**0.56 (0.46–0.68)**	**<0.001**	**334/2,489**	**569/2,455**	**0.61 (0.54-0.70)**	**<0.001**
**All-Cause Hospitalization/ER Visits**	**1,720 / 2,546**	**1,671 / 2,546**	**1.03 (0.97–1.11)**	**0.33**	**2,025/2,546**	**2,024/2,546**	**1.04 (0.97-1.10)**	**0.259**
**Stroke/CVA**	**78 / 1,638**	**88 / 1,543**	**0.83 (0.62–1.13)**	**0.243**	**185/1,638**	**221/1,543**	**0.83 (0.68-1.01)**	**0.058**
**Acute CHF**	**492 / 2,546**	**484 / 2,546**	**1.02 (0.90–1.16)**	**0.758**	**1,176/2,546**	**1,225/2,546**	**1.00 (0.92-1.08)**	**0.904**
**GI Symptoms**	**762 / 2,546**	**774 / 2,546**	**0.98 (0.88–1.08)**	**0.644**	**724/2,546**	**740/2,546**	**1.02 (0.92-1.13)**	**0.73**
**Atrial Fibrillation**	**48 / 771**	**42 / 770**	**1.18 (0.78–1.79)**	**0.432**	**96/771**	**94/770**	**1.16 (0.87-1.54)**	**0.315**

Supplementary Table S8. Outcome-specific denominators, prior event exclusions, and event counts in the propensity-matched cohort.

Outcome	Cohort	Matched Cohort (n)	Excluded for Prior Outcome Before Time Window (n)	Eligible Patients at Risk (n)	Events at 1 Year (n, %)	Events at 3 Years (n, %)
Primary Composite Outcome (AMI/Stroke/All-Cause Mortality/Acute HF)	Colchicine	28,941	15,281	13,660	2,313 (16.9%)	4,665 (34.2%)
	No Colchicine	28,941	15,943	12,998	2,577 (19.8%)	5,157 (39.7%)
All-Cause Mortality	Colchicine	28,941	910	28,031	3,182 (11.4%)	6,880 (24.5%)
	No Colchicine	28,941	1,728	27,213	4,008 (14.7%)	8,207 (30.1%)

Stroke/CVA	Colchicine	28,941	10,490	18,451	1,105 (6.0%)	2,307 (12.5%)
	No Colchicine	28,941	11,044	17,897	1,098 (6.1%)	2,418 (13.5%)
Atrial Fibrillation	Colchicine	28,941	17,231	11,710	910 (7.8%)	1,897 (16.2%)
	No Colchicine	28,941	17,729	11,212	841 (7.5%)	1,813 (16.2%)
Acute Heart Failure	Colchicine	28,941	0*	28,941	5,216 (18.0%)	7,972 (27.5%)
	No Colchicine	28,941	0*	28,941	5,060 (17.5%)	7,671 (26.5%)
All-Cause Hospitalization/ED Visits	Colchicine	28,941	0†	28,941	16,195 (55.9%)	20,251 (70.0%)
	No Colchicine	28,941	0†	28,941	15,943 (55.0%)	19,916 (68.8%)

*** Acute heart failure analyses included patients with prior heart failure diagnoses because the outcome specifically captured acute diastolic decompensation events (ICD-10 I50.31 and I50.33).**

† Hospitalization analyses were configured within TriNetX to include patients with prior hospitalization events before the start of the time window.

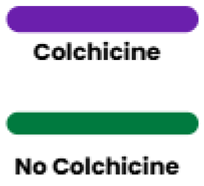

Supplementary Figure S1: Propensity score density function: before and after matching.

Note: Purple for the colchicine cohort and green for the no-colchicine cohort.

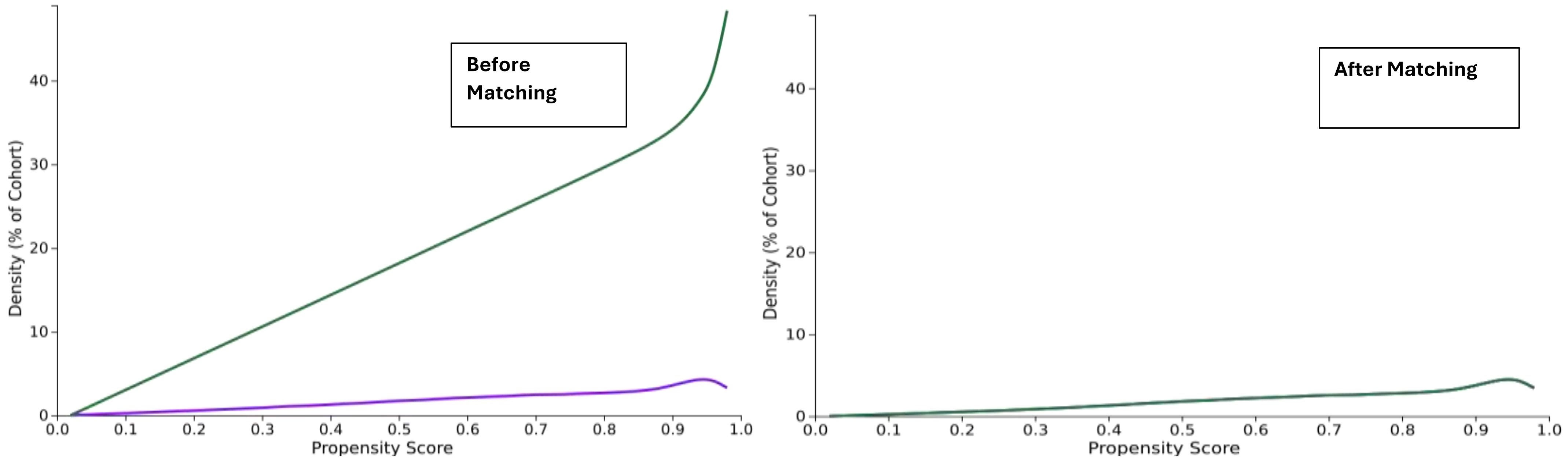

Before Matching
After Matching
Density (% of Cohort)
Propensity Score
0
10
20
30
40
0.0
0.1
0.2
0.3
0.4
0.5
0.6
0.7
0.8
0.9
1.0

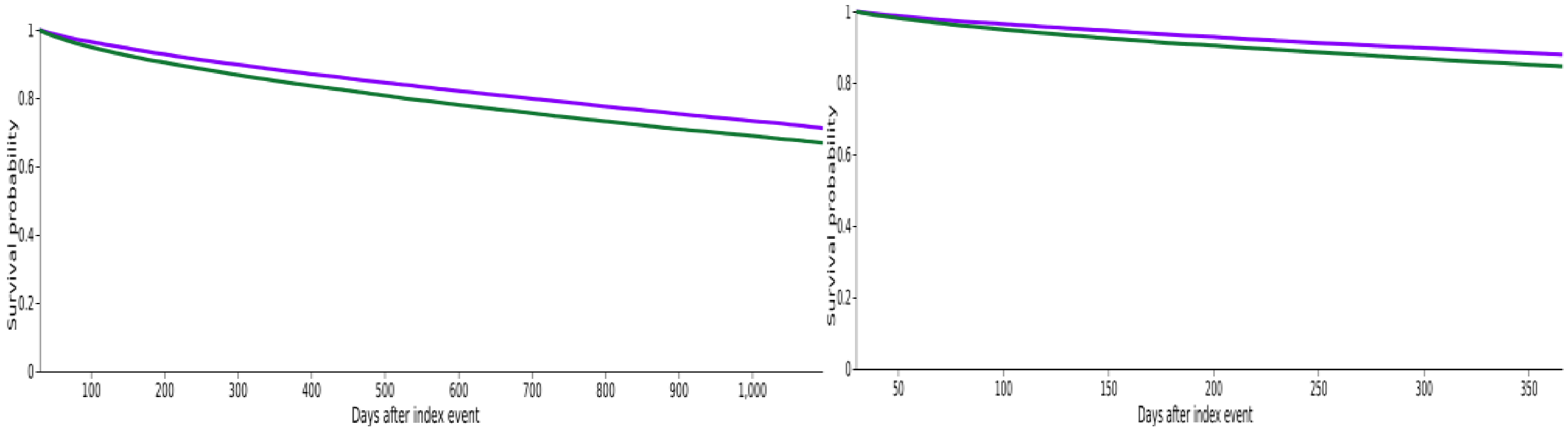

Supplementary Figure S2: Kaplan–Meier curves for all-cause mortality for 1-year and 3-year follow-up respectively.

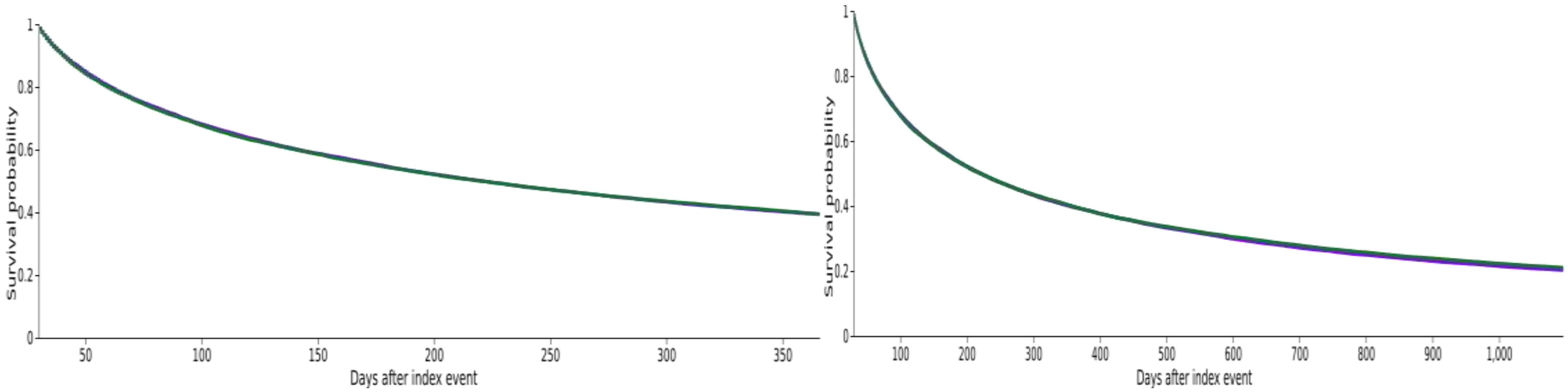

Supplementary Figure S3: Kaplan–Meier curves for all-cause hospitalization for 1-year and 3-year follow-up respectively.

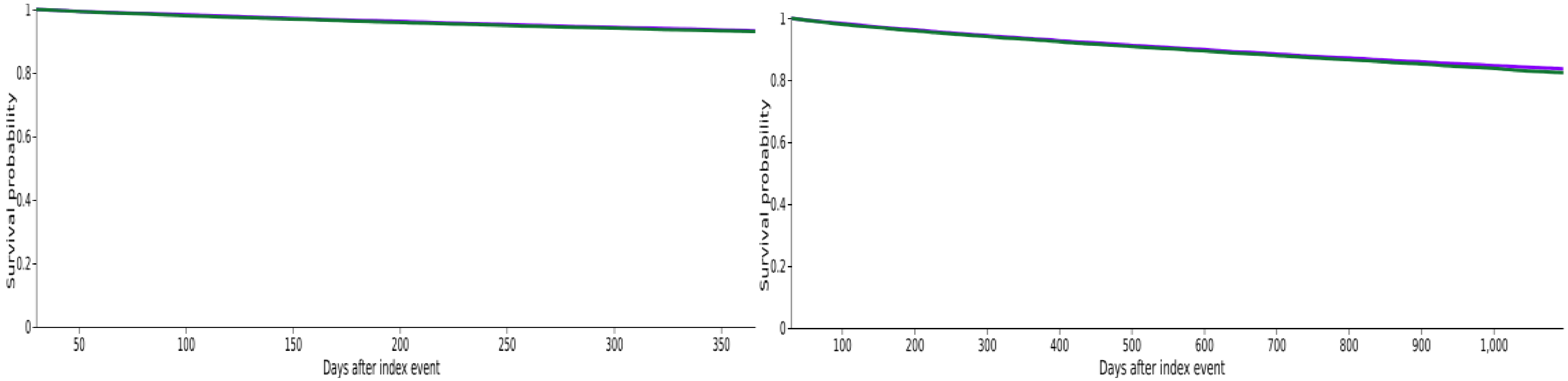

Supplementary Figure S4: Kaplan–Meier curves for stroke for 1-year and 3-year follow-up respectively.

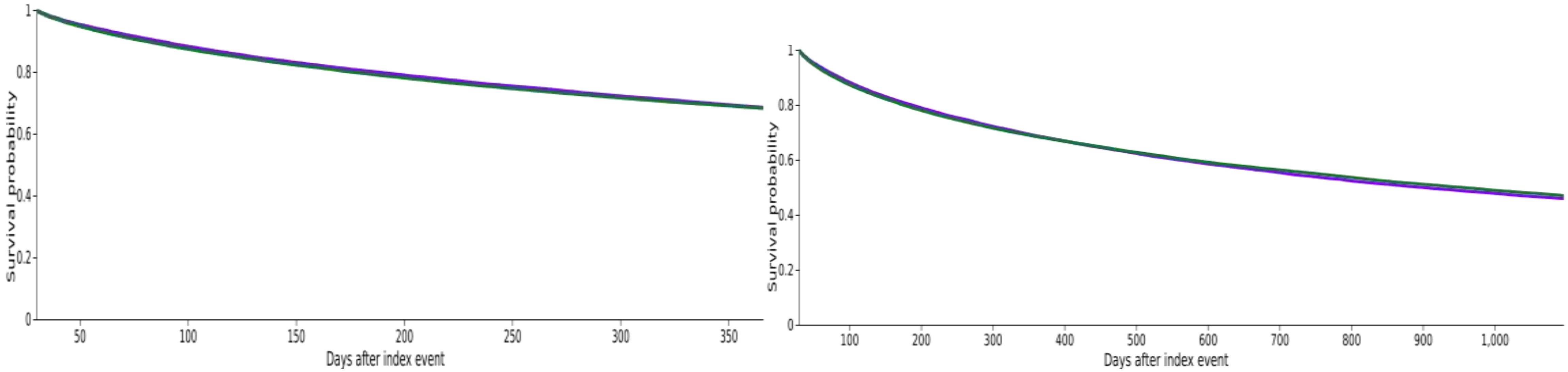

Supplementary Figure S5: Kaplan–Meier curves for GI symptoms for 1-year and 3-year follow-up respectively.

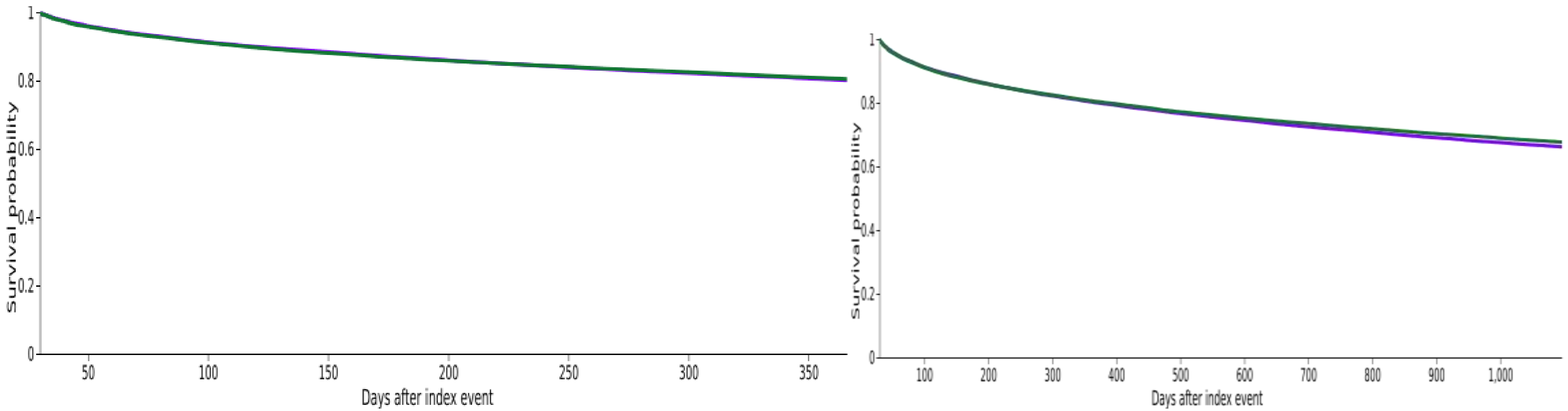

Supplementary Figure S6: Kaplan–Meier curves for acute CHF for 1-year and 3-year follow-up respectively.

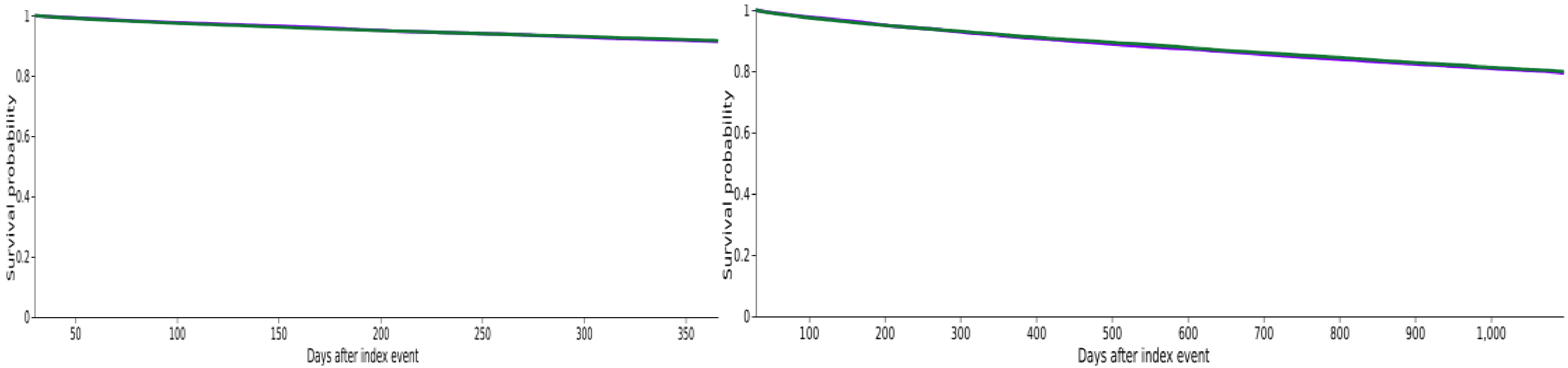

Supplementary Figure S7: Kaplan–Meier Curves for atrial fibrillation for 1-year and 3-year follow-up respectively.
